# Microintaglio Printing for Soft Lithography-Based *in Situ* Microarrays

**DOI:** 10.3390/microarrays4030311

**Published:** 2015-07-14

**Authors:** Manish Biyani, Takanori Ichiki

**Affiliations:** 1 Green Devices Research Center, School of Materials Science, Japan Advanced Institute of Science and Technology, 1-1 Asahidai, Nomi, Ishikawa 923-1292, Japan; 2 Department of Bioengineering, School of Engineering, The University of Tokyo, 2-11-16 Yayoi, Bunkyo-ku, Tokyo 113-8656, Japan

**Keywords:** microarray, intaglio printing, bioprinting, *in situ* synthesis, microreactor array

## Abstract

Advances in lithographic approaches to fabricating bio-microarrays have been extensively explored over the last two decades. However, the need for pattern flexibility, a high density, a high resolution, affordability and on-demand fabrication is promoting the development of unconventional routes for microarray fabrication. This review highlights the development and uses of a new molecular lithography approach, called “microintaglio printing technology”, for large-scale bio-microarray fabrication using a microreactor array (µRA)-based chip consisting of uniformly-arranged, femtoliter-size µRA molds. In this method, a single-molecule-amplified DNA microarray pattern is self-assembled onto a µRA mold and subsequently converted into a messenger RNA or protein microarray pattern by simultaneously producing and transferring (immobilizing) a messenger RNA or a protein from a µRA mold to a glass surface. Microintaglio printing allows the self-assembly and patterning of *in situ*-synthesized biomolecules into high-density (kilo-giga-density), ordered arrays on a chip surface with µm-order precision. This holistic aim, which is difficult to achieve using conventional printing and microarray approaches, is expected to revolutionize and reshape proteomics. This review is not written comprehensively, but rather substantively, highlighting the versatility of microintaglio printing for developing a prerequisite platform for microarray technology for the postgenomic era.

## 1. Introduction

**Figure 1:**
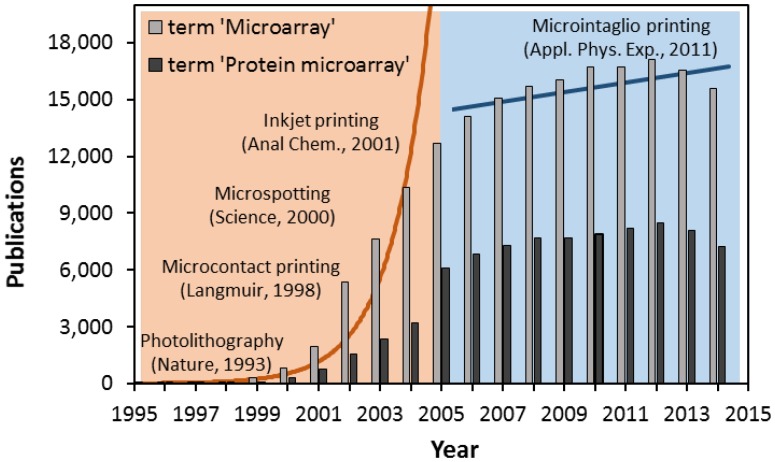
Growth in the number of publications on microarrays. The number of papers published in the last two decades utilizing the term “microarray” or “protein microarray” in the title (searched using Thomson Scientific Web of Knowledge) has increased continuously. The number in the first decade (1995–2005) increased exponentially. However, the increase was less significant in the second decade (2006–2014).

A microarray is typically defined as a collection of microscopic spots of biological solutions attached and arranged in a defined location on a solid surface that allows a massive number of parallel genotyping and/or phenotyping measurement. The fundamental principles of parallelized microspot (microarray) technology were conceived of on the basis of the ambient analyte model of Roger Ekins and colleagues in the late 1980s. However, the enormous interest evoked by microarray-based assays came in the late 1990s, when researchers were first able to utilize high-quality and reproducible results using DNA chips [1]. Since then, microarray technology, involving the direct transfer of biological molecules to a substrate using molecular printing techniques, has been extensively explored; there have been over 186,000 publications about the potential applications of microarray technology for scientific discoveries in a broad range of biological disciplines (Figure 1). The exponential growth in the number of publications in its first decade (1995–2005) has resulted in the maturation and potential application of microarray technology for biological research. However, the growth was less rapid in the following decade, known as the postgenomic era, which revealed the limitations, pitfalls and redesign considerations that must be addressed for the microarray approach to meet expectations in the field of proteomics [2,3]. Protein microarrays are not only emerging as an alternative tool to DNA chips to profile protein products, but are also expected to link both genomics and proteomics. The purpose of this review is to discuss the key innovations and technological limitations that must be overcome to develop a breakthrough platform for microarrays, which are no longer determined by technical advances, but by the expected experimental requirements.

**Figure 2:**
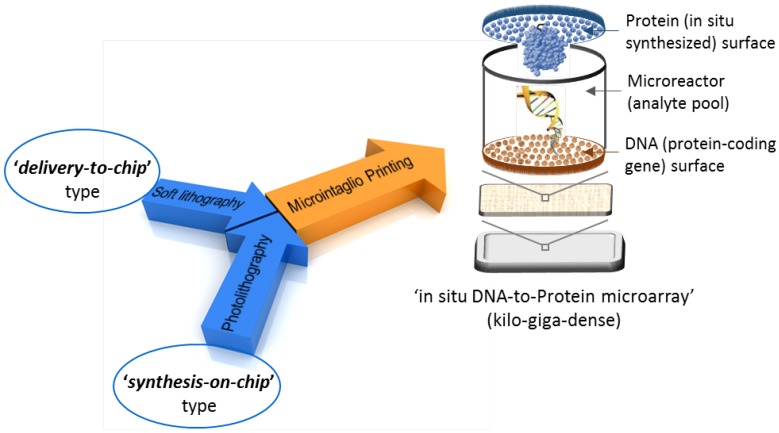
Key approaches in the historical development and future outlook of microarrays.

The breakthrough in the fabrication of microarrays came through the development of two parallel key approaches (Figure 2). The first was the “delivery-to-chip”-type approach using a robotic spotter, where biological molecules, such as DNAs, are dispensed by spotting and physically attaching presynthesized biological molecules to a solid substrate using robotic printing technologies. In this approach, source plates (such as microtiter plates with a 96- or 384-well format) are first filled with a presynthesized biological solution, which is followed by the transfer of a few nanoliters of the solution per spot onto the microarray chip surface using pins or piezoelectric dispensers to produce an array of submicrometer features [4]. The probe spots can be applied by either contact printing (e.g., microstamping [5] or microcontact printing [6]) or noncontact printing (e.g., microspotting [7], inkjet printing [8], laser writing [9], photochemical printing [10] or nanosphere lithography [11,12]), as reviewed in [13]. The key technical challenges in creating high-quality microarrays include the efficient transfer of biological molecules onto substrates and achieving a high coupling efficiency. Microspotting has been the most straightforward and leading platform, where biological molecules are synthesized off a chip then transfer-printed on a chip using a robotic spotter. A multi-pin mode (typically 12 × 4 pins) instead of a low-throughput single-pin mode can produce arrays in a highly parallel manner, but has limitations in terms of the size and density of arrays, because the pitch between pins must match the pattern of the microtiter plates. Therefore, the spotter-based robotic arraying approach is practically limited to producing low-speed and low-density arrays of spots. Second, this approach requires tedious and time-consuming multistep reactions to spot individual biomolecules from a separately-synthesized and purified solution followed by their immobilization on the surface of the array. The successive loading and dispensing of the biological solution are time-consuming and can lead to cross-contamination and even the loss of functionality of biological molecules, such as proteins, owing to denaturation or dehydration. Thirdly, robotic spotting has relatively high start-up costs and is thus less affordable. Core facilities typically charge $80–$300 per spotted array [14].

The soft lithography method developed by Whitesides’ group [15] is a complementary molecular printing technique. Specifically, microcontact printing (µCp), a form of soft lithography, has flourished as a simple, reproducible, cost-effective patterning method. In µCp, elastomeric stamps are prepared by pouring an elastomer, such as poly(dimethylsiloxane) (PDMS), in a mold. The polymer is then cured in the mold, thereby generating a relief structure in the elastomeric stamp. The stamp can then be used to directly transfer molecules to a surface of interest. PDMS polymer has the advantages of low toxicity, high flexibility and low cost. Although PDMS molds have been utilized with great success in preparing microarrays, alternative mold materials, such as cross-linked perfluoropolyether (PFPE), can be used with some distinct advantages over PDMS-based materials [16]. However, µCp also has several drawbacks. Although the stamps can print over large areas, they can only deposit a single predetermined pattern, and thus, a new mold must be fabricated each time for each printing pattern. Furthermore, nonuniformity is another major concern, as the patterning resolution is affected and limited by stamp swelling and shrinkage. In addition, µCp has limitations in printing multicomponent materials, such as bioarrays, and therefore, a convenient strategy for the massive patterning of biomolecules using soft lithography is yet to be developed.

The second major approach to microarray fabrication was the development of photolithography by Stephen Fodor and colleagues at Affymax Research Institute, which has made an important contribution to the fabrication of “synthesis-on-chip”-type microarrays [17]. In this approach, arrays are constructed *in situ* by actually producing the biological molecules directly on the substrate with extremely high density using photochemistry and solid-phase synthesis [17,18]. Affymetrix Corporation has been a pioneer in this field by developing the GeneChip^®^ [19]. Parallel on-chip gene synthesis has also recently been reported [20]. However, because of the complex nature of the chemical synthesis and the intrinsically expensive process involved in production, this method is usually inflexible and expensive. The capital investment required to build a clean room makes this method inaccessible to most researchers. Thus, this method is only feasible when applied to a limited set of materials. Therefore, the challenge to date has been to overcome the most pressing and ever-growing issue, *i.e.*, increasing the current low capacity (a typical density of thousands of array elements) of microarrays to and ultradense (mega-giga) capacity. As shown in Figure 2, the next forthcoming issue in microarray technology is the inclusion of a key feature that can facilitate a link between individual DNA sequences (genetic information) and their protein products (functional information) for gene expression profiling and related applications. To meet these requirements, we have recently introduced and developed a basic technology termed “microintaglio printing” (µIP) for next-generation, high-density microarray production [21,22]. This method is a conceptually different low-cost and spotter-free arraying approach to the rapid prototyping of microscale patterns, where biological molecules are intaglio-printed on a substrate using a microfabricated printing mold. Furthermore, the incorporation of our original work on fabricating high-density DNA microreactor array chips makes this approach very simple and robust for achieving high-density DNA-linked protein microarrays [23,24]. Therefore, the µIP approach can provide a direct link between recombinantly-expressed proteins and the corresponding DNA sequence information. Consequently, genetic information in clone libraries can be integrated with their biological functional information and potential interaction partners (Figure 2).

## 2. Concept of Microintaglio Printing

**Figure 3:**
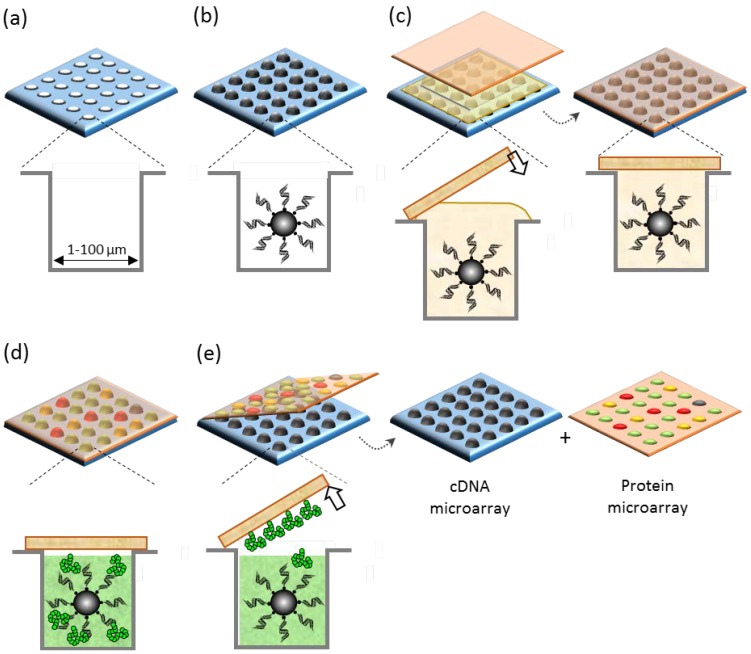
Schematic illustration of key steps in microintaglio printing (µIP) for the *in situ* fabrication of microarrays. A microengraved intaglio plate (micromold plate) consisting of a dense array of microchambers (**a**) is filled with the precursors of biomolecular “ink” (e.g., single-DNA molecule-amplified bead carrier or messenger RNA-immobilized bead carrier) (**b**); A glass substrate, one surface of which is modified to capture the biomolecular ink, is used to sandwich the cell-free system (e.g., coupled transcription/translation system or translation system) and seal the microchambers (**c**); The filled microchambers are placed in contact at a temperature for the *in situ* synthesis of the biomolecular ink within the microchambers (**d**); and the synthesized biomolecular ink (e.g., proteins) is then diffused and captured on the glass substrate (**e**). The assembly is peeled off to release the printed protein microarrays from the corresponding DNA microarrays.

This method is based on the principle of the gravure printing (roll-to-roll printing) process using a dense array of microengraved plates (micromold plates). Figure 3 outlines the major steps of the procedure used for µIP: (i) the fabrication of a microchamber-array chip; (ii) the self-assembly and arraying of biomolecular “ink”; and (iii) the *in situ* synthesis and patterning of microarrays.

### 2.1. Fabrication of Arrays of Microchambers

A microchamber-array chip is a basic requirement for soft lithography-based µIP. Micromold plates with desired features can be designed by computer software and prepared by photolithography or ordered from commercial suppliers. These plates are then used to fabricate arrays of microchambers using soft lithography by the replica molding of monolithic slabs of PDMS. To demonstrate the concept of µIP, microchambers of 1.5–100 µm in diameter and depth, giving a density of 40,000–20,000 uniformly-distributed independent microchambers per mm^2^, were employed.

### 2.2. Self-Organization and Arraying of Precursors of Biomolecular Ink

Biomolecular libraries, such as proteomes in a cell or tissue lysate, which are anticipated to be composed of over 100,000 proteins, exist as mixtures in a solution and must first be individualized and then organized into a one-to-one format prior to analysis using a microarray approach. This can be carried out by several techniques, including photolithography and contact printing. However, these are not high-throughput and robust ways of dealing with a full-length library. µIP uses a BEAMing (beads, emulsion, amplification, magnetic) approach [25] to first conduct a single-molecule PCR on a single magnetic micrometer-sized bead carrier using a water-in-oil emulsion. The construct of the DNA is modified to express the proteins with a double-histidine tag. These single-molecule-amplified DNA bead carriers are then self-aligned onto intaglio plates (PDMS micromold plates with dense arrays of microchambers) through an external dynamic magnetic force that is applied briefly by sliding a permanent magnet horizontally along the bottom of the micromold plates. The beads are arranged with only one bead in each microchamber owing to physical constraints in the design of the beads and microchambers. We have recently reported the rapid assembly of ultrahigh-density (144 million) DNA microbead arrays using a magnetic field [23,24,26].

### 2.3. In Situ Synthesis and Patterning of Microarrays

This step enables the co-synthesis and immobilization of biomolecular ink from individual microchambers arrays to corresponding positions on a glass substrate. A droplet of a cell-free-coupled transcription/translation system is sandwiched between a Ni-NTA-modified glass surface and a bead-incorporated microchamber array chip by applying pressure. To ensure perfect contact between the microchamber array chip and the glass substrate, the chip is vertically pressed onto the glass using a bench-mounted hand press on ice. Then, the coupled transcription/translation reaction is initiated by increasing the temperature from 4 °C to room temperature and incubating the assembly at 30 °C for 60 min. Multiple copies of a messenger RNA are transcribed from the bead-bound DNAs in the individual compartments of the microchamber-array chip, which is followed by the immediate translation of the double-histidine-tagged protein, which diffuses to the glass surface and is captured by the Ni-NTA-modified glass surface.

## 3. Microintaglio Printing-Based Biomolecular Microarrays

### 3.1. Printing and Arraying of Ready-To-Print Biomolecular Ink

RNA microarrays, such as RNA aptamer microarrays for the detection and quantization of protein biomarkers in biological samples, are a powerful tool for analyzing RNA-related phenomena. DNA microarrays are commonly used and fabricated by well-developed methodologies. However, a similar methodology may not be applicable to the fabrication of RNA microarrays, because of the extremely labile nature of RNA molecules. The previously reported methods of fabricating RNA microarrays were performed by the direct attachment of a messenger RNA onto a substrate after chemically-modifying the RNA using thiol-gold chemistry [27] or by mechanical transfer from a DNA master [28]. Recently, an on-chip enzymatic synthesis process has also been reported as a means of fabricating RNA aptamer microarrays in a microfluidic format [29]. These methods are successful when applied to a limited set of arrays, although the need for chemical modification is a concern. µIP enables the fabrication of high-density transcribed RNA microarrays with high throughput. In addition, since µIP enables the parallel *in situ* co-synthesis and immobilization of biological molecules, the need for chemical modification and the risk of degrading labile RNA can be reduced.

**Figure 4:**
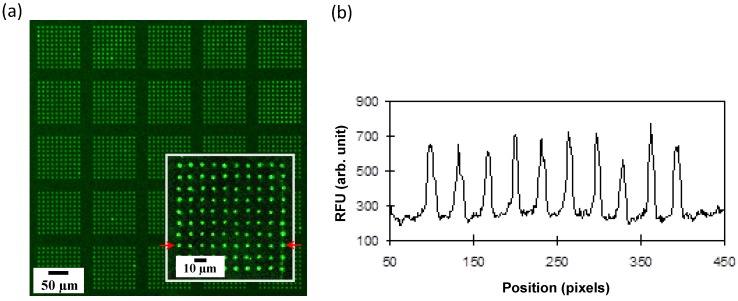
Printing and arraying of presynthesized transcribed RNA molecules. (**a**) Microscopy image of the printed pattern of full-length messenger RNA with 5-µm array features using the µIP approach; (**b**) Fluorescence distribution plot for a single row of patterned messenger RNA (indicated by the red arrow in (**a**)). RFU, relative fluorescence units.

The first demonstration of µIP was the successful patterning of a 5-µm-diameter spot of presynthesized messenger RNAs at a density of 10,000 per mm^2^ [21]. Figure 4 shows a fluorescence micrograph of a 5-µm-scale array pattern of the full-length transcribed messenger RNA acquired by confocal laser scanning microscopy. As can be seen in Figure 4a, high background noise (*i.e*., low contrast between the foreground (spots) and background) is observed, which is approximately 250 RFU (relative fluorescence units), *i.e.*, nearly one-third of the actual signal intensity. This suggests that the printing of ready-to-print ink molecules (*i.e*., presynthesized messenger RNAs in this case) must be improved for high-resolution patterning using the µIP approach. Note that there is a risk of part of the ink molecules (*i.e*., messenger RNA molecules in this case) remaining in the non-array region (outside the grooves on the printing mold) while achieving a tight seal between the microchamber-array chip and the substrate. Thus, the ink molecules remaining in contact with the non-array region before being forced out by the sealing step may also be undesirably printed in the non-array region on the substrate, leading to high background noise or low-contrast printing.

**Figure 5:**
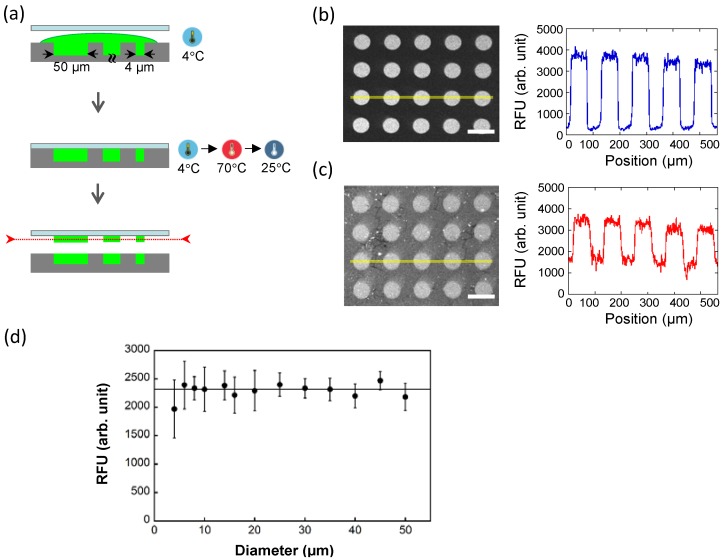
Advanced temperature-controlled microintaglio printing (TC-µIP). (**a**) Schematic of TC-µIP. Biomolecular ink is first applied on a printing mold, and the mold is pressed with a capturing probe-modified substrate at a low temperature. The binding interaction between the biomolecular ink and the probe, which is suppressed at a low temperature, can then be activated by temperature control; (**b**) Microscopy images and printed patterns of full-length messenger RNA molecules with (**b**) and without (**c**) using the TC-µIP approach. The signal/noise ratios were calculated from the intensity along the yellow line in each image; (**d**) Fluorescence intensities per pixel of fluorescence-labeled messenger RNA spots with several diameters using the TC-µIP approach. The printed intensities were comparable from diameters of 4–50 µm. Error bars represent the mean ± SD (*n* = 9–24 spots). The scale bar indicates 100 µm.

To accomplish high-contrast µIP, we have recently introduced an advanced form of µIP by controlling the adsorption and/or printing reaction of the presynthesized ink molecules via a thermal trigger (Figure 5) [30]. This is based on a controllable hybridization reaction between a presynthesized messenger RNA and a substrate-immobilized complementary DNA probe by switching the ability of the messenger RNA to fold into a secondary structure via the temperature during µIP processing. At a low temperature, the messenger RNA remains in a folded state, in which it is unable to hybridize, whereas a high temperature can convert the folded state into the unfolded state required for the denaturation of the secondary structure of messenger RNA molecules, thus inducing the hybridization reactions. Therefore, temperature switching before and after the press-sealing step in the µIP process can prevent the undesired printing of presynthesized messenger RNA molecules in the non-array region (Figure 5a). Using this method, we reported the fabrication of transcribed RNA microarrays with a diameter of 1.5 µm and a density of 40,000 spots per mm^2^ with high contrast (Figure 5b,c) [30]. Most importantly, we also demonstrated that the uniformity of patterned signals over a range of microarray feature sizes spanning one order of magnitude is not affected, and thus, this method can be used for the miniaturization of arrays to obtain high-density nanoarrays. As shown in Figure 5d, a printing mold having microchambers with various diameters of 4–50 µm and similar heights generated pattern intensities with a comparable efficiency.

### 3.2. Printing and Arraying of In Situ-Synthesized Biomolecular Ink

**Figure 6:**
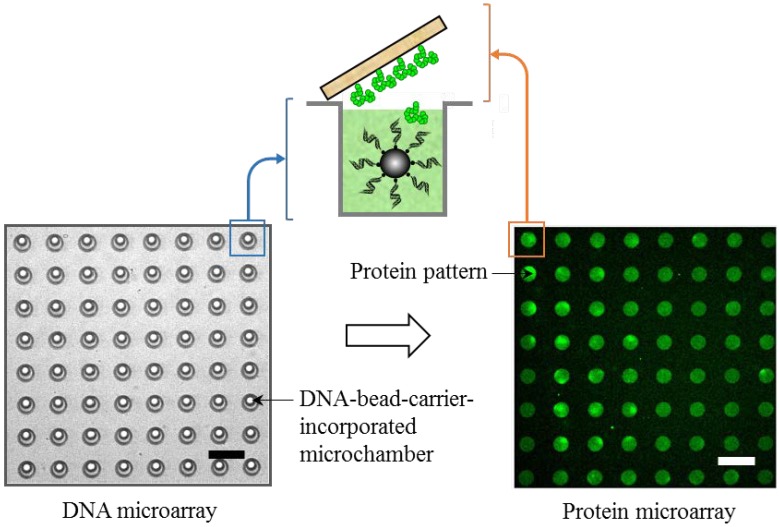
Printing and arraying of *in situ*-synthesized protein molecules. Bright-field icroscopy image of DNA-bead carrier-incorporated microreactor array chip (**left image**) and fluorescence microscopy image of *in situ* co-synthesized and patterned GFP spots (**right image**). The scale bar indicates 100 µm.

The limitations of both the leading technologies historically employed to manufacture microarrays, *i.e.*, the synthesis-on-chip approach using photolithography and the delivery-to-chip approach using microcontact printing, have made it impractical to broaden the applicability of microarrays in the postgenomic era. Photolithography is still a very expensive and time-consuming process, and microcontact printing is restricted to a limited number of arrays and also to the deposition of presynthesized biomolecular ink. Although DNA microarrays have achieved commercial success using these approaches, it is important to note that protein microarrays have not been successful, since they are crucially dependent on maintaining the complex 3D quaternary structure of proteins on the microarray surface, which is technically difficult using these conventional approaches (Figure 1). Therefore, the concept of the on-demand *in situ* creation of microarrays is giving a new meaning to the term ‘custom’ microarrays, which greatly reduce the risk of loss of function when arraying biomolecular ink and also remove the need for handling steps, such as purification and modification, prior to printing. For this purpose, previously, the concept of a DNA-linked protein array was presented, where we adopted a strategy of one-to-one indexing between spatially unknown individual DNA arrays and their encoded protein array products *in situ* [31,32]. Next, this process was miniaturized by demonstrating the advanced application of µIP for the *in situ* co-synthesis and printing of protein microarrays on demand. First, magnetic beads carrying a dsDNA sequence encoding double-histidine-tagged GFP were arrayed onto a microchamber array chip. In the next step, a droplet of a cell-free-coupled transcription/translation system was sandwiched between a Ni-NTA-modified glass surface and a bead-incorporated microchamber array chip. Then, the coupled transcription/translation reaction was initiated by increasing the temperature from 4 °C to room temperature and incubating the assembly at 30 °C for 60 min. Figure 6 shows that histidine-tagged GFP was successfully synthesized *in situ* from the bead-bound template DNA inside the microchamber of the microchamber-array chip and transferred to the Ni-NTA-modified glass substrate to produce a clear 60-µm-scale array pattern that was successfully detected using a confocal laser microscope [22]. These results confirm the successful one-step *in situ* synthesis and printing of individual proteins from localized DNAs and that this approach can be used to rapidly create large-scale integrated protein microarrays directly from the encoded DNA microarrays.

Although a single protein was arrayed and printed in the above demonstration, in principle, this concept is extendable to the simultaneous printing of multiple proteins per array using BEAMing and self-assembled magnetic bead approaches [23]. This has been demonstrated by fabricating kilo-giga-density DNA microarrays [24] and the on-chip synthesis and arraying of a randomized library of mutant GFP using an ultrahigh-density (144 million) microbead array format [26]. Therefore, the inclusion of an *in situ* synthesis process in µIP enables the development of a beyond-mega-spot protein microarray format that can be used not only to express whole proteomes on a chip, but also to create novel artificial proteins by massively increasing the repertoire of analyzable proteins up to the level used in molecular-directed evolution.

### 3.3. Instrument-Free Arraying of “Kilo-Giga”-Dense Microarrays with High Resolution

The printing of higher density protein microarrays is highly desirable for global proteome analysis. Similarly, ultrahigh density DNA or RNA microarrays are a prerequisite for on-chip novel DNA or RNA probe (*i.e*., aptamer) screening purposes, which deal with huge aptamer libraries with up to 10^15^ variants. Conventional microarrays fabricated by a robotic spotter or a photolithographic approach have a number of immobilized spots on a single array substrate that is limited to the 10^5^ order. However, µIP can be considered as an ultrahigh-throughput printing tool for the instrument-free and inexpensive printing of arrays of biomolecules. Owing to the fundamental nature of µIP, this technology would allow the printing of spotter-free ultra-small microarrays while maintaining a high resolution and, thus, can overcome the low-medium-density limitations of current methods. Using µIP, we demonstrated the printing of *in situ*-transcribed messenger RNAs onto part of a 30 mm × 30 mm glass substrate with a density of 40,000 spots per mm^2^, which to our knowledge, is the highest ever density reported for microarrays. To demonstrate the high-resolution characteristic of µIP, the printing of an arbitrary shape, a pattern comprising the letters “RNA” with a line width of 7.5 µm, was also achieved [31]. Therefore, µIP technology is considered to provide an instrument (spotter)-free platform for generating *in situ*-synthesized biomolecular (messenger RNA/protein) microarrays with an ultrahigh density (kilo-giga scale) and a high resolution (sub-micrometer).

## 4. Outlook and Future Direction of µIP

The development of µIP has provided fundamental insight into: (1) the spotter-free arraying of ready-to-print biological molecules; (2) the *in situ* co-synthesis and immobilization of messenger RNA or protein molecules directly from prearrayed DNA; (3) the self-assembly of microarrays with an increased density of up to 25 million per chip, which is 3–4 orders of magnitude higher than that of existing approaches; and (4) high resolution with sub-micrometer features. Despite the success of µIP, several aspects of printing must be improved. First, the current protocol of µIP requires the use of a microchamber-array chip fabricated by top-down microfabrication using soft lithography, which relies on the use of photolithography to generate the master, which is not a universally-affordable technique. In an advanced form of µIP, it may be possible to produce the microchamber-array chip by solution processing, *i.e.*, a solution-based deposition technique, which will help significantly to reduce the cost and increase the availability of µIP. Second, a PDMS-based microchamber-array chip is used for µIP; the limitations of the use of PDMS in cell-free protein synthesis-based microarrays are its propensity to adsorb analytes and its porosity, which allows small molecular components of the cell-free system, such as amino acids, to diffuse into the bulk of PDMS [33]. Minimizing the scale, which increases the surface area of PDMS relative to the sample volume, can thus alter arraying outcomes, as a result of interfacial phenomena. Recently, we have investigated the effectiveness of a PDMS surface for miniaturizing the cell-free *in situ* synthesis of GFP inside PDMS microchambers with different sizes, and thus, different surface area-to-volume (S/V) ratios, and observed that the yield in the cell-free synthesis of GFP in PDMS microchambers decreased monotonically with decreasing microchamber diameter from 100 down to 25 µm [34]. Therefore, the application of coating techniques to suppress the PDMS interfacial phenomena can increase the suitability of PDMS while obtaining the benefits of µIP for developing ultrahigh-density protein microarrays. In summary, µIP can be applied to simultaneously synthesize and pattern virtually any full-length protein at a micrometer-scale resolution from prearrayed DNAs, and thus, this system is a versatile platform for several applications, including the on-chip molecular screening of mutant protein libraries, the identification of novel protein sequences and the detection of protein to protein interactions for global proteome analysis.
